# Assessing Clinical Impressions of Early Warning Score Integration With the Rapid Response Team: Protocol for a Prospective Cohort Study

**DOI:** 10.2196/65360

**Published:** 2025-07-31

**Authors:** Alexandre Tran, Rashi Ramchandani, Jamie Brehaut, Natasha Hudek, Jessica Haines, Irene Watpool, Rebecca Porteous, Dora Kusevic, Kirby Bucciero, Kwadwo Kyeremanteng, Michael Hartwick, Kednapa Thavorn, Jonathan Hooper, Dalibor Kubelik, Christophe Herry, Nathan Scales, Brett Hryciw, Jad Abou-Khalil, Jeffrey Perry, Christopher Bredeson, Andrew Seely

**Affiliations:** 1 Division of General Surgery Department of Surgery Ottawa Hospital Ottawa, ON Canada; 2 Department of Critical Care Ottawa Hospital Ottawa, ON Canada; 3 Acute Care Research Ottawa Hospital Research Institute Ottawa, ON Canada; 4 Department of Medicine Faculty of Medicine University of Ottawa Ottawa, ON Canada; 5 Methodological and Implementation Research Ottawa Hospital Research Institute Ottawa, ON Canada; 6 Division of Palliative Medicine Department of Medicine Ottawa Hospital Ottawa, ON Canada; 7 Department of Emergency Medicine University of Ottawa Ottawa, ON Canada; 8 Division of Hematology Ottawa Hospital Ottawa, ON Canada; 9 Division of Thoracic Surgery The Ottawa Hospital Ottawa, ON Canada

**Keywords:** early warning score, Visensia safety index, rapid response team, intensive care unit, critical care, resuscitation

## Abstract

**Background:**

The use of early warning scores (EWSs), which integrate real-time vital sign monitoring, can help rapid response teams (RRTs) proactively identify patients at risk of deterioration. However, existing EWSs demonstrate limited evidence for the reduction of clinically important adverse events. The Visensia Safety Index (VSI) is an EWS that combines heart rate, blood pressure, temperature, oxygen saturation, and respiration rate vital sign information to generate a VSI acuity score ranging from 0, signifying the lowest risk of deterioration, to 5, signifying highest risk of deterioration. Continuous monitoring of the risk of deterioration, with alerts triggered by a score of 3.0 or greater, prompts medical attention.

**Objective:**

This protocol outlines the methodology for assessing the feasibility of combining a portable continuous vital sign monitoring system (Masimo Root Monitor) with VSI monitoring to evaluate patients at high risk of acute deterioration at The Ottawa Hospital (TOH).

**Methods:**

This 2-phase, prospective cohort study will be conducted at TOH. Patient participants will include adults (≥18 years) with high-risk conditions, such as those undergoing high-risk elective surgery, malignant hematology or oncology patients, and those admitted with infections. Exclusion criteria include patients receiving comfort care or those in specialized units requiring higher-level monitoring. Eligible patients will be monitored using the VSI with alerts to trigger rapid response team evaluation. In tandem, health care workers including physicians, nurses, and research staff who are involved in patient care and monitoring will be recruited for semistructured interviews. These interviews will explore health care providers’ clinical impressions of the VSI as an EWS to identify barriers and enablers to implementation using the Consolidated Framework for Implementation Research. Interviews will assess impressions of the monitoring technology, clinical workflow challenges, and perceptions of implementation. Data analysis will involve directed content analysis of interview transcripts to identify barriers and drivers for successful implementation. Furthermore, the feasibility of the VSI will be assessed by evaluating the proportion of continuous data successfully collected and transmitted, the timeliness of VSI-triggered alerts and their communication to the RRT team, the regular updating of the predictive tool, the rate of reported VSI-triggered events, and the completion rates of study-related tasks by research and clinical staff.

**Results:**

As of April 2025, we can report that enrollment is complete, and analysis of the results is ongoing. Interviews are still being transcribed and analyzed. We anticipate submission for publication of the results of the study by the summer of 2025.

**Conclusions:**

The execution of this prospective, mixed methods feasibility study requires a multidisciplinary effort. The study uses continuous vital sign monitoring, combined with prediction of deterioration risk, used to activate the RRT. Evaluating the feasibility and clinical impressions of this trial implementation is the first step in exploring monitoring-based predictive decision support.

**Trial Registration:**

ClinicalTrials.gov NCT05108376; https://clinicaltrials.gov/study/NCT05108376

**International Registered Report Identifier (IRRID):**

DERR1-10.2196/65360

## Introduction

Many hospitals have adopted rapid response teams (RRTs) that often include registered nurses (RNs), respiratory therapists, and physicians who provide urgent critical care expertise to assist with managing unstable patients outside of the intensive care unit (ICU) [1,2]. Early warning scores (EWSs) incorporate routinely collected data such as vital signs or laboratory results to provide a systematic, evidence-based approach for identifying vulnerable patients for assessment before a critical deterioration event [3]. Although numerous EWSs exist, many are based on a single vital sign, thereby relying on a single vital sign crossing a static threshold without accounting for trends over time or the interplay between multiple physiologic changes. For example, tools like the National Early Warning Score or Between-the-Flags primarily use fixed criteria to detect abnormalities, which can result in low sensitivity and missed detection of complex deterioration events [4]. This is in line with the traditional and current metric for monitoring deterioration with vital signs often monitored intermittently, with abnormal values flagged manually or through basic thresholds in electronic medical records [5,6]. However, this approach can delay the identification of clinical deterioration, as it may not account for trends over time or the combined significance of multiple vital sign abnormalities [6,7]. Importantly, delayed detection of patient deterioration has been associated with adverse outcomes, including higher rates of ICU admission, prolonged hospital stays, cardiac arrest, and mortality [8,9]. Recent evaluations of single vital sign–based EWS tools like the Queensland Adult-Deterioration-Detection-System and the electronic Cardiac Arrest Risk Triage Score (eCART) highlight variability in predictive accuracy, with eCART demonstrating superior sensitivity but also higher alerting rates, which can overwhelm clinical workflows [10]. Additionally, while EWS and RRT systems are widely implemented, evidence of their effectiveness in reducing hospital mortality, unplanned ICU admissions, and other adverse outcomes remains limited and of low certainty, underscoring the need for standardized outcome measures and improved methodologies in future studies [11].

**Figure 1:**
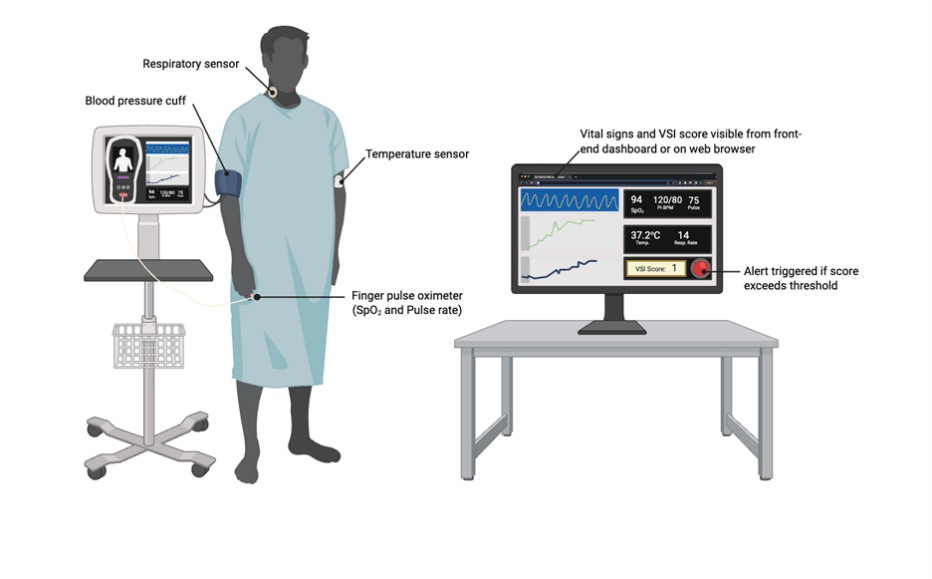
An infographic showing the patient setup for this study. Image created on BioRender. SpO2: oxygen saturation; VSI: Visensia Safety Index.

In contrast to relying solely on a single vital sign for cues of deterioration, OBS Medical’s Visensia Safety Index (VSI) is an EWS that uses physiological monitoring software and machine learning to identify the likelihood of deterioration that may lead to cardiac or respiratory arrest [3,5]. As depicted in Figure 1, the VSI is a continuous measure that combines 5 vital signs—heart rate, blood pressure, temperature, oxygen saturation, and respiratory rate—into a single acuity score ranging from 0 (lowest risk) to 5 (highest risk). This score is recalculated in near real-time, with updates occurring at one-minute intervals as new vital sign data are collected. A score of 3.0, or a predetermined threshold determined based on clinical context, or greater represents a critical threshold, suggesting a significant risk of clinical deterioration and prompting an alert to the RRT [4]. The dynamic nature of the VSI allows for continuous monitoring of patient stability and early identification of deterioration trends, enabling timely interventions. Unlike traditional Early Warning Scores, VSI dynamically integrates multiple parameters into a single metric, enabling earlier and more precise detection of deterioration trends. While all five vital signs contribute to the VSI calculation, the system can operate with a minimum of three input parameters, ensuring functionality even when some data are unavailable. This capability makes VSI particularly robust for continuous patient monitoring in dynamic clinical environments.

A retrospective cohort study of 168 patients found that 95% of the episodes of severe physiological abnormalities were identified by the VSI system and deemed valid, defined as requiring a change in clinical management [12]. A 3-phase prospective study was implemented for validation at the University of Pittsburgh Medical Centre, which included a continuous collection of cardiorespiratory variables in a non-ICU patient population [13]. Patients monitored with VSI had a statistically significant shorter average duration of any instability, shorter average duration of physiological instability, and fewer episodes of serious and persistent instability [13]. While continuous monitoring likely contributes to these outcomes, the VSI integrates multiple physiological parameters into a single predictive score, enabling earlier identification of deterioration trends compared to traditional EWS. By providing a more comprehensive and dynamic view of patient stability, VSI facilitates timely clinical interventions that may not occur with traditional monitoring systems alone [4].

A recent retrospective cohort study at The Ottawa Hospital (TOH) compared outcomes for patients who would have had an EWS alert, triggered by VSI or the National Early Warning Systems 2 (NEWS2), prior to RRT activation to patients without an EWS alert [14]. It was found that an EWS would have identified earlier deterioration for over half of the patients; however, clinical impact requires interventional study. While both the VSI and NEWS2 may have contributed to the favorable findings of early deterioration detection, EWS like NEWS2 have limitations in sensitivities, underscoring the need for more dynamic tools like VSI to improve early detection in real-world settings [5,15].

Studies involving multiparameter EWS, like the VSI, have shown promise in identifying patient deterioration earlier than clinical judgment alone, much of the existing work has been limited to proof-of-concept or validation studies, which do not address the complexities of real-world implementation. The integration of such tools into hospital workflows remains challenging due to the numerous moving parts required for successful adoption, including technical barriers, clinician buy-in, and alignment with existing clinical processes. These barriers underscore the critical need for feasibility studies that evaluate not only whether the system can be implemented within a hospital context, but also how its integration could be improved by incorporating feedback from healthcare providers. This study offers a protocol designed to address these gaps by taking a focused approach to feasibility: assessing the practicality of implementation at a single institution and identifying key barriers and enablers to facilitate smoother adoption at other sites.

The objectives of this study are to evaluate the initial clinical impressions and feasibility of using a portable monitoring system with a VSI trigger. More specifically, feasibility will be assessed by examining the proportion of continuous data successfully collected and transmitted, the frequency and timeliness of VSI-triggered alerts, the regular updating of the predictive tool, and the completion rates of study-related tasks. Clinical impressions will be explored through qualitative interviews with physicians, nurses, and research staff, focusing on barriers and enablers to implementation, perceived strengths and limitations of the system, and its integration into clinical workflows (Table 1). Overall, this protocol paper seeks to outline the methodology for evaluating the feasibility of integrating a portable continuous monitoring system using the VSI and exploring clinical impressions of its implementation to identify barriers, enablers, and potential clinical impact.

**Table 1:** Description of the study’s primary objectives of feasibility and impact as well as how each will be evaluated and monitored.

Evaluation component	Objective	Objective description	Evaluation of objective
Evaluation	Feasibility	Evaluate the practicality of continuous data collection, continuous data feeding, regular updating of the predictive tool, and communication with the RRT^a^.	This study will be considered “technically feasible” if 80% of (a) continuous data are collected, (b) continuous data are fed to the predictive tool, (c) the time the predictive tool is updated on a regular basis, and (d) of triggers are reported to the RRT team.
Evaluation	Barriers and enablers to implementation	Perform a qualitative analysis of physicians, nurses, and research personnel regarding impressions of the predictive tool, perceived strengths and limitations, and perceived enablers and barriers to effective usage.	Physician, nurse, and research personnel opinions regarding impressions of the predictive tool, perceived strengths and limitations, and perceived enablers and barriers to effective usage will be evaluated through theory-driven postencounter interviews.
Impressions	Clinical impact	Compare the predictive performance of the VSI trigger to clinical gestalt.	Clinical impact will be assessed by the patient acuity rating [15], and performance measures including sensitivity, specificity, positive predictive value, and negative predictive value.

^a^RRT: rapid response team.

## Methods

### Overview

This study uses a mixed methods design to comprehensively evaluate the feasibility of implementing a portable continuous monitoring system with VSI-triggered alerts. The quantitative component focuses on assessing feasibility outcomes, such as data collection and transmission rates, VSI alert timeliness, and completion rates of study tasks. The qualitative component involves semistructured interviews with healthcare providers to explore their clinical impressions, including barriers, enablers, and perceptions of usability and integration into workflows. Together, these complementary approaches aim to provide a holistic understanding of the implementation process.

### Study Setting

This study will be conducted at both the Civic Campus and the General Campus of TOH. Each campus has a tertiary-level, mixed medical-surgical ICU with a 32-bed capacity that uses the same RRT activation criteria and institutional database, although each caters to a different patient population. Specifically, patients at the General are typically from the hematology, medical oncology, and thoracic surgery wards, whereas those from the Civic are trauma, neurology, neurosurgery, or vascular cases.

The criteria for RRT activation were defined as concerns relating to the airway, consciousness, cardiovascular system, or other markers of deterioration as deemed by clinical discretion. Data for the RRT database will be prospectively gathered by the responding RN using a standardized data collection form at the time of patient assessment.

#### Eligibility Criteria

Selection criteria for patient participants and VSI users who will participate in the subsequent semistructured interviews are outlined in Table 2. The study population will include patients outside of units with high-level monitoring, such as ICUs, as these areas already have continuous vital sign monitoring systems in place. Eligible participants will be adult patients (≥18 years) admitted to general medical or surgical wards across the two hospital campuses, which each have mixed medical-surgical ICUs with 32 beds. Patients categorized as “Category 1–Full Care” will be included as they will have no limitations on critical care interventions. Patients with “Category II, III, and IV” statuses are excluded due to goals of care that prioritize comfort or limited interventions, which do not align with the proactive monitoring and management strategies being evaluated in this study (Table 2).

**Table 2:** Inclusion and exclusion criteria for patients and clinicians.

Criteria	Inclusion	Exclusion
Patient participants	Adult patients (≥18 years of age) designated for the most aggressive levels of potential intervention (Category 1 status–Full Care) belonging to any of the following: Patients who have undergone high-risk elective surgery. Malignant hematology or oncology patients at high risk for deterioration. Patients with infections admitted from the Emergency Department to the ward. Other high-risk patients determined at the discretion of RRT^a^ physicians with agreement from the patient’s MRP^b^.	Patients admitted to a unit with higher-level monitoring, including the Neurological Acute Assessment Unit, Acute Monitoring Area, Trauma Step-Down, or Intensive Care Unit. Patients in Category II, which includes full care except for CPR^c^, Category III which includes full care except respiratory or hemodynamic life support, or CPR or category IV status, which is focused solely on Comfort Care.
Visensia Safety Index users	Physicians, RNs^d^, and research personnel who consent to participate in an interview and are the enrolled patients: RRT physician or ward physician. RRT RN or ward RN. Research personnel.	Physicians, RNs, or research personnel unable to complete the interview in English.

^a^RRT: rapid response team.

^b^MRP: most responsible physician.

^c^CPR: cardiopulmonary resuscitation.

^d^RN: registered nurse.

#### Design

**Figure 2:**
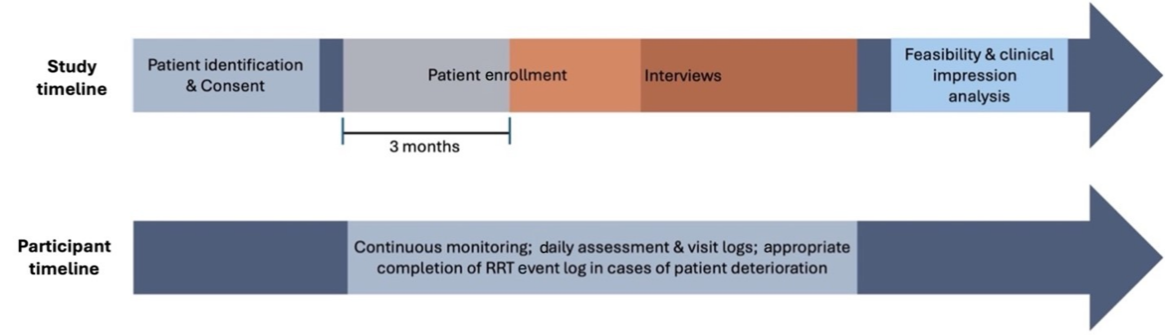
Visual overview of study timeline and design with emphasis on overlap in study timeline with semistructured interviews beginning 3 months after patient enrollment. RRT: Rapid Response Team.

This study uses a prospective, mixed methods feasibility design. The enrollment and monitoring involve the patient’s most responsible physician, and members of the RRT including physicians, RNs, or respiratory therapists. Eligible patients will be identified based on the inclusion criteria listed in Table 2 and approached for potential consent. During the initial interaction, research staff will discuss the study protocol, review eligibility criteria, and assess willingness to participate. Written consent will be obtained from patients prior to initiating monitoring, A study summary is depicted in Figure 2 highlighting the study timeline and steps followed in this study.

#### Monitoring

After enrollment, the patient will be connected to a vital sign monitor (VSM). The Masimo Root monitor, a portable continuous monitoring device that integrates multiple physiological parameters, including oxygen saturation, pulse rate, and respiration rate, will be used in this study. For this study, the device is equipped with a continuous monitoring license, which allows it to perform uninterrupted data collection and enable the calculation of VSI scores. This ensures seamless monitoring and timely alert generation for high-risk patients. Research staff or the RRT RN will ensure that the patient’s data output is visible at the RRT monitoring station set up in the ICU. The monitoring system will send vital signs data to the VSI vital sign platform (VSP) server, which will process the data and calculate the VSI score, which will appear at a monitoring station set up in the ICU. VSI notifications will also be available on a mobile device carried by RRT members.

Of the 5 vital signs monitored, a minimum of 3 are required by the VSI to calculate a patient’s acuity score. To ensure accuracy, all components measuring vital signs must be appropriately connected to the patient and the VSM. As depicted in Figure 1, for the Masimo Root monitor, the patient has (1) a temperature sensor patched above the cubital fossa, (2) a finger pulse oximeter measuring oxygen saturation and pulse rate on the index finger, (3) a blood pressure cuff on either arm that intermittently inflates, and (4) a respiratory sensor placed to enable auscultation of breathing laterally to the thyroid cartilage. The RRT physician and RN can view and monitor all enrolled patients and their VSI index via the VSP front-end dashboard, which can be accessed via a standard web browser or approved mobile devices. For the Masimo VSM, viewing acuity scores across devices is only possible if the VSM is connected to a local Wi-Fi network. A visual setup of the study can be seen in Figure 1.

#### Patient and Study Team Timeline

Once the patient is on continuous monitoring, the following will be completed on a daily basis:

1. The patient is clinically assessed in person by the RRT, as per the standard of care for patients on the RRT roster. These assessments will include a clinical evaluation of the patient’s condition, a review of vital sign trends, and any alerts generated by the VSI.
2. The RRT RN completes the Daily RRT visit log to capture the opinion of deterioration risk for the ward and RRT staff and the status of VSI monitoring. This includes the nurse’s assessment of the patient’s risk of deterioration based on clinical judgment, observations of vital sign trends, and any relevant changes in the patient’s condition.
3. The RRT RN completes the RRT event log for any calls to the RRT for patients.
4. The research coordinator completes the patient’s case report form, capturing demographic data as well as any deterioration events and outcomes.

#### Response Team Activation

The RRT RN will clinically assess a patient on the inpatient ward if an RRT call is (1) activated traditionally at the clinical discretion of the MRP team or RN or (2) activated by the VSI trigger. Importantly, VSI does not directly lead to clinical decision-making. Clinical decision-making will depend upon the assessment by the practitioners on the RRT.

In this study, the VSI critical threshold is defined as ≥3.0, which is an indication to practitioners of potential deterioration, defined as being a rapid and negative decline in monitored vital signs that require clinical monitoring or intervention. When the VSI reaches ≥ 3.0, the RRT is signaled for activation. This threshold may be changed during the study to balance sensitivity and specificity appropriate to the target population and clinical context. Adjustments will be made based on real-time observations of system performance and clinical relevance, aiming to ensure optimal detection of deterioration while minimizing false alerts. Any changes to the threshold will apply to all enrolled patients to maintain consistency.

The RRT RN will call or visit the ward to discuss the patient’s potential deterioration if a VSI alert is triggered. If erroneous or artefactual data are suspected, the bedside and RRT RNs will troubleshoot the monitoring issue with support from research staff as required. If deterioration is suspected, an RRT member will visit the patient and initiate a formal RRT call if warranted. Patient participation in the study will be complete when continuous monitoring is no longer required, following a minimum of 48 hours and up to a maximum of 10 days. Patients will be followed via their electronic medical records until hospital discharge. Importantly, the maximum monitoring period of 10 days was determined based on the study’s data storage and management capabilities, as this is a pilot study to evaluate the feasibility of using VSI acuity scores. Monitoring duration for each patient will be decided collaboratively by the research team and clinical staff, considering the patient’s clinical condition and study requirements. A future study could evaluate the long-term use of VSI scores beyond this threshold to assess its utility.

#### Interviews

Incorporating a portable continuous monitoring system with a VSI trigger into practice constitutes a complex intervention [7,9]. Therefore, we will conduct postimplementation semistructured interviews with involved physicians, RNs, and research personnel members of the RRT and wards where the portable continuous monitoring with VSI trigger is implemented. Importantly, despite not being part of the clinical team, research personnel were involved in setting up the study, including backend management of the VSI acuity score generation and ensuring proper functionality of the monitoring systems used in the study. Their expertise with the technical aspects of the VSI system and its integration into workflows makes their insights valuable in evaluating the ease of implementation and identifying potential barriers or enablers. Including them in interviews allows for a comprehensive assessment of the implementation process from both technical and clinical perspectives.

Interviews will be 30 to 45 minutes in anticipated length and structured using the Consolidated Framework for Implementation Research (CFIR) [16-18]. This framework describes the range of the barriers and drivers to the effective implementation of complex interventions at multiple levels and among many stakeholders; it has been used in health care settings to evaluate the implementation of innovations [17,19,20]. We will select CFIR constructs relevant to the current implementation collaboratively with the research team. We will develop questions relevant to each selected construct to explore the range of barriers and drivers to using the Masimo monitoring and VSI trigger–induced alerts and evaluate health care providers’ intentions to use such EWS in clinical practice. The guide will be pilot-tested for length, clarity, and comprehensiveness and revised as needed.

Potential interviewees will be identified by the research team and emailed an initial invitation and consent form to participate, with up to 2 email reminders. Verbal consent will be obtained at the time of the interview. Interviews will be divided into 2 sections. Section 1 will include background and demographic questions. Section 2 will assess potential barriers and drivers to using the monitoring and alerts according to the CFIR, with particular attention to (1) questions about the monitoring itself and how it compares to current processes, (2) environmental issues around resources and the setting in which the monitoring is being used, (3) the individuals involved in the implementation and other colleagues that may assist with or impede use, and (4) the implementation process itself including the implementation context and the adaptability of the innovation and setting. We will also ask about goals for use, and beliefs about outcomes related to monitoring use (see Multimedia Appendix 1 for the full interview guide including related CFIR constructs). Interviews will be audiotaped and transcribed, and field notes taken during the interview. All physicians, RNs, and research personnel who participate in an interview will receive a token of appreciation for their time and expertise.

### Sample Size

The sample size is based on a convenience sample obtainable with the currently available resources. The target feasibility metrics for this study are depicted in Table 1. Projected enrollment is 1-2 new patients per week at both centers for a total of 70 patients over a 10-month period. The sample size of 70 patients was selected to ensure sufficient variability in patient characteristics and clinical scenarios to evaluate feasibility outcomes, including the proportion of continuous data successfully collected and transmitted, VSI alert timeliness, and completion rates of study-related tasks.

Approximately 10-15 physicians, and 10-15 nurses, from both the RRT and ward will be interviewed alongside 2-4 research personnel who are involved in the care of patients enrolled in the clinical implementation portion of this study. Purposive sampling will include consideration of all patient subgroups listed in the inclusion criteria to ensure respondents are from a variety of clinical backgrounds. The interview sample size estimates are based on previous work around theme saturation using theory-informed interviews [21].

### Data Management

The output of VSI is comprised of two components: (1) the first produces a real-time risk score based on the patient’s own vital signs to ultimately send alerts to clinicians and (2) a user interface with all patients and their acuity continuously displayed and updated. The system has current US Food and Drug Administration (FDA) and Conformité Européenne (CE) clearance.

The VSI is a software application consisting of both a software backend hosted on a hospital server and a front-end dashboard accessible via a web browser. It is backed by two databases: a low-volume database storing the configuration information and a high-volume database storing anonymized vital sign information. Data are fed into the VSP by means of Health Level 7 (HL7), version 2. Feeds communicated once every minute provide a good balance between data storage requirements and data usefulness. They will be correlated to the same anonymous identification number as the vital sign data.

Several options exist for the anonymization of HL7 data from patients, depending on the hospital infrastructure. In this study, the anonymization will be performed on the VSP server via the installation of Mirth Connect before sending the data to the VSP. The VSP’s own database (anonymized vital signs) would be accessible by the sponsor (for study management purposes); however, the mapping table between study IDs and Protected Health Information/Personally Identifiable Information would only be accessible by the study data custodian.

#### Safety Reporting

Any safety issues related to the VSI will be addressed by the study co-principal investigators and study sponsor, OBS Medical. RRT RNs and physicians can bring any safety concerns to the RRT leads, who will communicate these to the study co–principal investigators. Concerns will be discussed and addressed on the same day. All safety issues will also be communicated to OBS Medical. Clinical issues will be addressed by the study co–principal investigators and technical issues will be addressed by OBS Medical. All safety issues will additionally be reviewed during the next monthly steering committee meeting.

All adverse device effects need to be assessed by the site investigator for severity, seriousness, expectedness, and causality/relatedness. The site investigator is required to report serious adverse events or serious adverse device effects to Health Canada and the Regulatory Correspondent within 72 hours of discovery. This includes cases in which the incident relates to the improper functioning or failure of the device or serious deterioration or death of the patient participant. It is expected that any serious untoward medical occurrence, unintended disease or injury, or untoward clinical signs (including abnormal laboratory findings) in subjects, users, or other persons, whether related to the investigational medical device, will be reported.

#### Study Management

A steering committee comprised of all investigators will meet monthly to discuss the study’s progress. This committee includes representatives from TOH leaders from information services, biomedical engineering, research coordinators, and investigators. Concerns raised by any staff will be evaluated monthly to guide changes to the protocol in an ongoing fashion. Interim results from interviews will be reviewed at every second meeting.

#### Data Analysis

Table 3 outlines the statistical analysis that will be conducted to evaluate the study objectives.

The study will collect both quantitative and qualitative data to address its objectives. The results evaluated will include outcomes listed in Textbox 1.

Descriptive statistics will summarize feasibility outcomes. Categorical variables will be presented as counts and percentages, and continuous variables as means and SDs (if normally distributed) or medians and interquartile ranges (if nonnormally distributed).

**Table 3:** Summary of statistical analysis.

Outcome domain	Objective	Statistical analysis
Evaluation	Feasibility	Representation of categorical variables as counts and percentages. Representation of continuous variables with either (1) means with SDs if a normal distribution is followed, or (2) medians with interquartile ranges if a non-normal distribution is followed.
Evaluation	Clinical impressions	Interview transcripts will be examined to identify barriers and enablers for the implementation of a VSI^a^ trigger using content-directed coding into CFIR^b^ constructs [8,9]. Additional inductive analysis of quotations within CFIR constructs for interpretation, with themes being periodically examined for revision, combination, and deletion as needed [12]. All transcripts will be double coded by 2 independent researchers meeting for consensus regularly.
Impressions	Clinical impact	Calculation of sensitivity and specificity with respect to the ability of the VSI score to predict intensive care unit admissions. Analysis of practitioner’s initial impressions of the system as well as challenges and benefits to its use.

^a^VSI: Visensia safety index.

^b^CFIR: Consolidated Framework for Implementation Research.

**Feasibility outcomes.**

**Feasibility outcomes**

- The proportion of continuous data successfully collected, transmitted, and analyzed using the Visensia Safety Index (VSI) system.
- The frequency and timeliness of VSI-triggered alerts and rapid response team activations.
- Completion rates for study-related tasks, including data logging, daily assessments, and interviews.
- Technical barriers encountered during system implementation and integration.

### Ethical Considerations

This study was approved by the Ottawa Health Science Network Research Ethics Board (OHSN-REB; protocol ID number 20210667-01). Written informed consent will be obtained from all participants, including patient participants and health care providers involved in interviews. Participants will have the ability to withdraw from the study at any time without any consequences. Privacy and confidentiality will be maintained by anonymizing all collected data, including interview transcripts, and storing data securely on encrypted servers accessible only to authorized study personnel.

### Clinical Impressions

Semistructured interviews with physicians, nurses, and research personnel will explore perceived barriers and enablers to implementing the VSI-triggered system. Directed content analysis into CFIR constructs will be used to identify barriers and drivers to use the monitoring and alerts in practice [17,18]. Coding according to CFIR allows for implementation-related barriers and drivers to be categorized into a widely understood and clearly defined series of constructs that can be acted upon for improved innovation uptake. For further interpretability of results, we will develop themes within identified CFIR constructs inductively, during consensus meetings between coders. Two reviewers will code all transcripts independently, meeting for consensus to agree on initial and new themes and revise, combine, and delete themes collaboratively.

We will take a reflexive approach to analysis, taking into consideration team members’ subjectivity to shape our qualitative methods and conclusions [22]. By having implementation experts from a separate department conduct the interviews we avoid power dynamics that could affect how interviewees respond and remain objective through coding and analysis. Coders have several years of experience in implementation science and qualitative methods and will meet with the wider study team regularly to ensure clinical relevance and credibility of results by involving a diverse team while maintaining objectivity. In using the CFIR framework to guide interviews and coding, we limit the influence of any one individual, while still capitalizing on the experience and expertise of the study team by enhancing the interpretability and applicability of CFIR constructs with inductively developed themes within the constructs. We have also considered methodological reflexivity in adding more general questions about any other issues and general inquiries about the overall impressions of the technology, which are also reflected in our coding plan, ensuring we do not limit responses and theme generation to only include CFIR constructs. Qualitative results will be analyzed alongside the quantitative feasibility data in designing future implementation plans.

### Predictive Performance

The predictive performance of the VSI-triggered alerts will be compared to clinical gestalt using sensitivity, specificity, positive predictive value, and negative predictive value. Quantitative data will be analyzed using statistical software, and subgroup analyses will account for variability across patient populations and clinical settings [21].

### Integration and Safety Reporting

Observations of system usage, including technical or clinical safety concerns, will be documented and analyzed. These findings will inform recommendations for future refinements to the monitoring system and its clinical deployment.

## Results

As of April 2025, we can report that enrolment is complete, and analysis is ongoing of the results. Interviews are still being transcribed and analyzed. We anticipate submission for publication of the results of the study by the Summer of 2025.

## Discussion

### Overview

This study aims to evaluate a trial implementation of a protocol to integrate continuous vital sign monitoring, predictive risk assessment for patient deterioration, and RRT activation. This initial implementation of the Masimo Root Monitor with OBS Medical’s VSI lays the groundwork for future developments in the field. By evaluating the feasibility of this integrated approach, we aim to participate in the early adoption of artificial intelligence in clinical decision support systems in acute and critical care environments [23]. However, the logistical challenges of collating real-time multidimensional data streams are noted to be a common limitation often ignored during initial validation efforts [23]—an important consideration that this feasibility study is intentionally designed to evaluate. We hypothesize that the integration of the VSI with a portable continuous monitoring system will demonstrate feasibility in clinical workflows and provide valuable insights into its potential to enhance early detection of patient deterioration, streamline RRT activations, and improve clinical decision-making processes.

### Comparison to Prior Work

Prior studies, including proof-of-concept work and retrospective analyses have demonstrated the potential of VSI, to identify patient deterioration earlier than traditional methods [3]. Notably, a three-phase prospective study at the University of Pittsburgh Medical Centre showed that integrating continuous monitoring with VSI reduced instability duration and improved patient outcomes [3,4]. However, these studies focused on validation, while this protocol emphasizes feasibility and implementation within real-world workflows at a Canadian tertiary care hospital.

### Anticipated Strengths and Limitations

This study’s strengths include its use of a mixed methods design, allowing for a comprehensive evaluation of both quantitative feasibility metrics and qualitative clinical impressions. Including a diverse, multidisciplinary team, who meet regularly to discuss study progress, share insights, and document decisions and challenges throughout the study process will improve the credibility, transferability, dependability, and confirmability of results. The use of the CFIR ensures a structured approach to understanding the barriers and enablers of implementation.

A notable limitation is that this study evaluates the feasibility of integrating the VSI within a continuous monitoring system to enhance RRT workflows; however, it does not isolate the independent effects of continuous monitoring, which may itself contribute to improved outcomes or operational efficiencies. Future studies will be required to differentiate the impact of continuous monitoring alone from the added value provided by predictive tools like VSI.

While conducting this study, we anticipate learning from individual patient deployments, particularly in addressing technical requirements and troubleshooting potential issues in integrating the VSI trigger with vital sign monitoring. For instance, as depicted in Figure 1, an acoustic respiratory sensor will be used in this study which requires adequate placement and limited environmental interference (patient talking and vibrations) to be registered and used in the calculation of a VSI acuity score. Moreover, the VSM must be connected to the hospital’s Wi-Fi to sync a patient’s real-time recorded vitals and their respective VSI to the VSP front-end dashboard that can be viewed by ward or RRT staff via a web browser. Training of research personnel, RRT, and ward staff on the use and maintenance of the VSM with a VSI trigger will allow for an eased integration and use of the system. Another anticipated limitation includes, despite its proof-of-concept work in the early phases of evaluating VSI feasibility and identifying barriers and enablers to implementation, practitioners involved in the care of patients in the study must appreciate that the VSI score must be contextualized by understanding the magnitude and trend of the index as well as the clinical context. We will evaluate clinicians’ experience of the monitoring and alerts in this study.

Health care provider perspectives regarding the clinical usefulness and utility of the VSI-based trigger will be evaluated during the semistructured interview portion of this study. It is well-known that the “black-box” nature of algorithms may limit acceptance by the medical community, especially when incongruent with clinical intuition [23]. As such, efforts to measure what matters most to patients and clinicians using evaluation frameworks from implementation science may be particularly valuable [24]. Due to the interdisciplinary nature of implementing such a device, a comprehensive framework is required to understand the barriers and enablers of its use, ways in which it can improve the efficiency and workflow of allied health care professionals within the team, identify necessary behavioral changes to overcome obstacles, and enhance the effectiveness of outcome measurement and comprehension. Such results can be achieved by following CFIR, a meta-theoretical framework that helps practitioners evaluate the implementation process and understand key factors that can impact implementation success. Using CFIR will allow the research team to design a comprehensive implementation plan to minimize barriers and leverage drivers for the integration of the VSI-based alerts and related monitoring more broadly into practice.

A multidisciplinary research team is essential to enable clinical and technical feasibility. The implementation of a VSM with a VSI trigger requires a coordinated effort by research personnel, external partnering agencies, physicians, RNs, and respiratory therapists part of RRTs and on the wards. As such, it is vital for all involved members to become familiar with the technical requirements of the portable VSM being used, the predictive algorithm of the VSI, the data management platform, and the VSP dashboard. Moreover, practitioners must be knowledgeable and trained in evaluating VSI-related clinical information on a daily basis with appropriate logging and reporting in the case of deterioration events.

### Future Studies

Following the completion of this feasibility study, subsequent research should focus on refining the integration of the VSI into routine clinical workflows. This includes assessing its impact on patient outcomes, such as reducing ICU admissions and preventing adverse events, through a larger interventional trial. Additionally, studies could explore the scalability of this system across hospital settings, including resource-limited environments, and investigate the incorporation of advanced machine learning algorithms to enhance predictive accuracy. Long-term evaluations should also examine cost-effectiveness and sustainability to support widespread adoption.

**Conclusion**

This study protocol outlines a mixed methods approach to evaluate the feasibility and clinical impressions of integrating continuous VSM with the VSI for early detection of patient deterioration and rapid response team activation. By combining prospective data collection with theory-informed qualitative interviews, this study aims to identify key implementation barriers and enablers within real-world hospital workflows. Findings from this feasibility study will inform the design of future interventional trials and guide the broader integration of predictive monitoring systems into acute care settings.

## Appendix

Multimedia Appendix 1 Interview guide.

## Abbreviations

CE: Conformité Européenne
CFIR: Consolidated Framework for Implementation Research
eCART: Cardiac Arrest Risk Triage Score
EWS: Early Warning Score
FDA: US Food and Drug Administration
HL7: Health Level 7
ICU: intensive care unit
NEWS2: National Early Warning Systems 2
RN: registered nurse
RRT: rapid response team
TOH: The Ottawa Hospital
VSI: Visensia Safety Index
VSM: vital sign monitor
VSP: vital signs platform

## Data Availability

Data sharing is not applicable as no datasets were generated or analyzed during the current study. As this manuscript outlines a study protocol, no datasets were generated or analyzed at this stage.
