# Supplementary material for: Assessing Clinical Impressions of Early Warning Score Integration With the Rapid Response Team: Protocol for a Prospective Cohort Study
Source: JMIR Res Protoc. 2025 Jul 31;14:e65360. doi: 10.2196/65360 (PMC12355135; doi:10.2196/65360)
Supplement: Multimedia Appendix 1 [file resprot_v14i1e65360_app1.docx]

# Masimo/Visensia Index Interview Guide

**INSTRUCTIONS:** ~ 2 minutes

Moderator: Ensure that the participant has received the Consent form

**INTRODUCTION SCRIPT FOR MODERATOR:**

*Thank you for agreeing to meet with us. As you may know, we are interested in implementing a new technology called the* ***Masimo monitoring and*** ***Visensia Index****, designed to help identify patients at high risk of deterioration who might need acute critical care. We are interested in your thoughts about the usefulness of this tool, how we can make it more usable.*

*We are going to be talking to RACE and ward physicians and nurses about the tool; we’ll be asking questions about your recent experience with the Masimo monitoring and Visensia Index alerts and technology.*

*Before we get started, we should mention a few things:*

- *I’m not a clinician, and so may need to ask you to clarify any jargon or clinical issues that are discussed.* *You are the experts in this discussion; we are interested in learning about your experience here.*
- *You can decide you’d rather not answer any particular question. If for any reason you’d rather not answer a question, no problem.*
- *Everything you say is completely private. Your name will not be mentioned in any report, your comments will only ever be reported anonymously, and your survey answers will only ever be reported in aggregate.*
- *We appreciate your suggestions. There are no right or wrong answers to any of our questions. We would like to hear all feedback, both positive and negative, about your experience with the technology we’ll be discussing.*
- *We are recording this session by audiotape. This will help ensure we learn as much as possible from these sessions. Is this OK with you?*
- *Another member of our team may listen in on the sessions, again to help us learn as much as possible from them. Is this OK with you?*

**********************

*ANY QUESTIONS SO FAR?*

***********************

*Ok…let’s get started.*

**SECTION 1: Icebreaker/demographics**

1. **What is your job title?**
2. **Are you on the RACE team or on the floor?**
3. **How many years have you been in practice?**
4. **How would you describe your gender?**

**SECTION 2: Questions about Masimo/Visensia Index technology and alerts**

*Now I’d like to ask about your experience(s) with the Masimo/Visensia Index alerts and technology.*

1. **Do you recall recently providing care to any patients who were being monitored using Masimo and Visensia Index technology? (If ‘no’ read the following description of Masimo/VSI: *Visensia index is an early warning score intended to inform health care providers that a patient’s condition is worsening and in need of a RACE team to be called. The monitoring consists of continuous monitoring performed by a Masimo ROOT monitor, and combined with routine vital signs data. The Visensia Index will provide auditory and visual display alerts to clinicians’ phones if a patient’s monitoring data indicates a worsening condition.)*Interface for WARD staff:


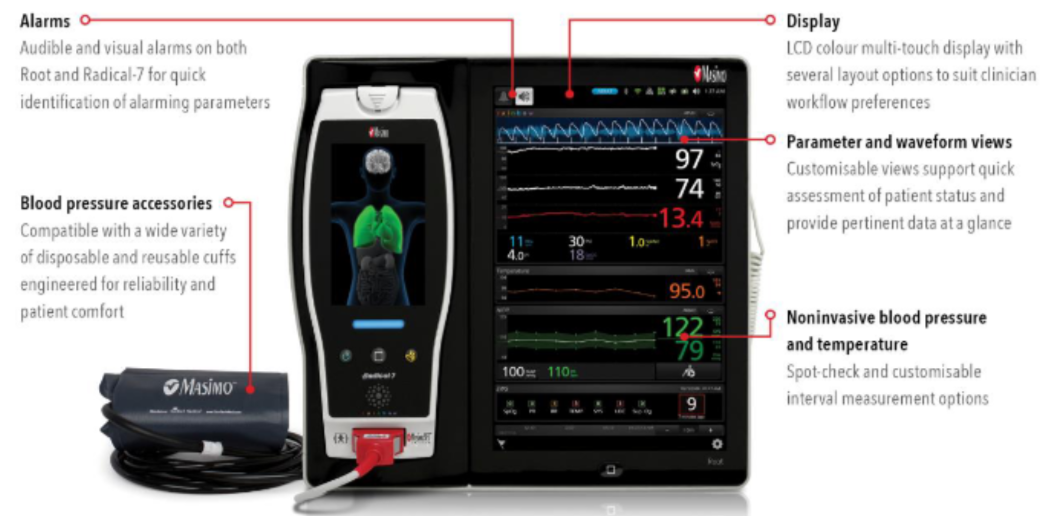


   Interface for RACE staff:


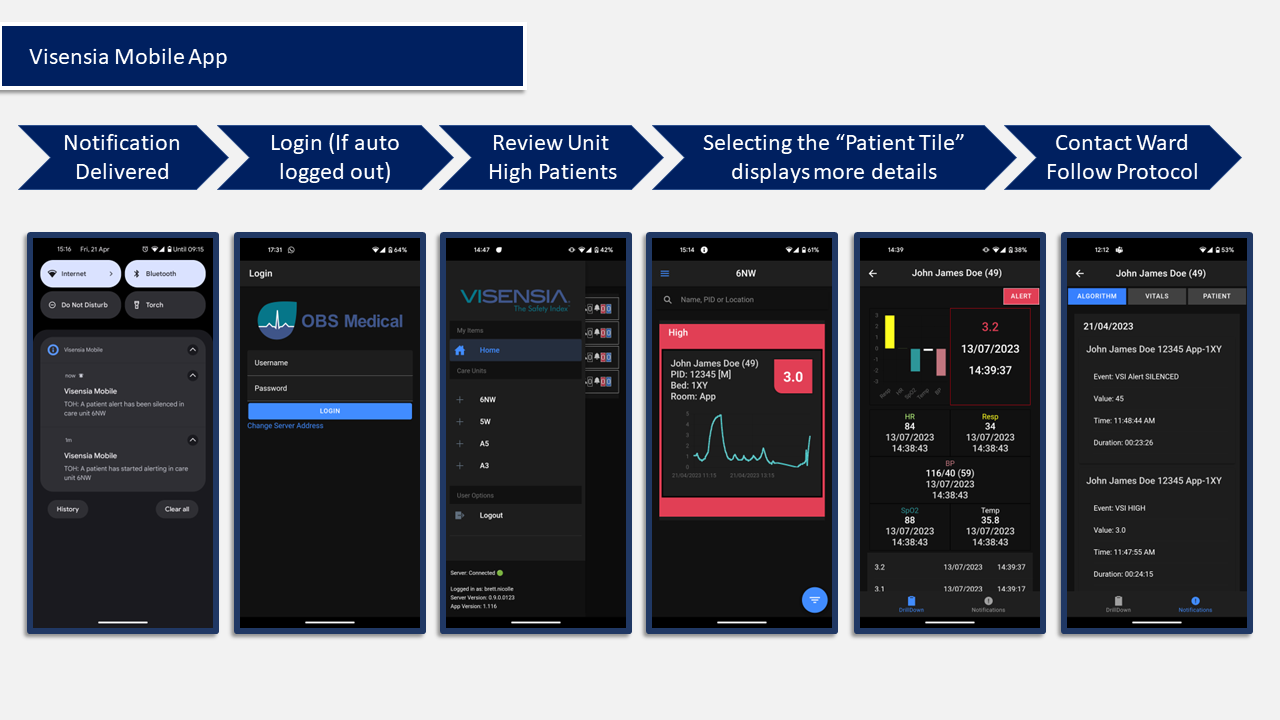
**
   1. **If ‘yes’, could you tell me a little about this (these) patient encounter(s)?**
2. **What is your overall impression about the [*Masimo or Visensia Index*] monitoring technology?**
3. **How does the [*Masimo or VSI*] monitoring compare to the current RACE call process in your setting? (CFIR Relative Advantage)**
4. **(*ward staff*) How complicated is the Masimo monitoring to set up and maintain? (CFIR Complexity)**
    **(*RACE team*) How complicated are the VSI predictive monitoring and alerts to understand? (CFIR Complexity)**
5. **What is your overall impression of the [*Masimo or VSI*] monitoring and alerts effectiveness? (CFIR Evidence Strength & Quality)**
6. **Have you encountered any false alarms or incorrect values? (CFIR Evidence Strength & Quality)**
7. **(*ward staff*) How will the physical set-up and resources within your unit affect the implementation of the Masimo monitoring? (CFIR Work Infrastructure)**

**(*RACE team*) How will the physical set-up and resources in the hospital affect integration of the VSI alerts into your current process? (CFIR Work Infrastructure)**

1. **How do you usually communicate patient needs with the RACE/ward team members? (CFIR Communications)**
2. **How would the [*Masimo or VSI*] monitoring and alerts affect the way you communicate with RACE/ward team members? (CFIR Communications)**
3. **Can you think of anything about the hospital’s culture that would affect the implementation of the [*Masimo or VSI*] monitoring? *(Culture: shared values, beliefs, and norms across the hospital)* (CFIR Culture)**
4. **Does the VSI/Masimo monitoring impact the hospital’s mission to provide patient-centered care in any way? (CFIR Recipient-centeredness)**
5. **How well does the [*Masimo or VSI*] monitoring fit with existing work processes and practices in your setting? (CFIR Compatibility)**
6. **Can you describe how the [*Masimo or VSI*] monitoring and alerts would best be integrated into current processes? (CFIR Compatibility)**
7. **Are you aware of any goals set related to the implementation of the [*Masimo or VSI*] monitoring? (CFIR Mission Alignment)**
   1. Prompt: Goals you set yourself? Set by your unit? The study team? The hospital?
8. **How confident are you that you will be able to use the [*Masimo or VSI*] monitoring? (CFIR Capability)**
9. **Do you have the skills and knowledge to use the [*Masimo or VSI*] monitoring effectively? (CFIR Capability)**
10. **How motivated are you to use the [*Masimo or VSI*] monitoring/alerts for inpatients? (CFIR Motivation)**
11. **Do you feel supported in using the [*Masimo or VSI*] monitoring? If so, by who? (CFIR Implementation Facilitators)**
12. **What might get in the way of the [*Masimo or VSI*] monitoring/alerts being used in practice? (CFIR Assessing Context)**
    1. Prompt: Anything else?
13. **Is there anything that would increase use of the [*Masimo or VSI*] monitoring/alerts among you and your colleagues? (CFIR Assessing Context)**
14. **Are there any modifications we could make to the monitoring or alerts to make it easier to use? (CFIR Adapting)**
15. **Are there are modifications we could make to the workflow or environment that would make the [*Masimo or VSI*] easier to use? (CFIR Adapting)**
16. **Do you think the [*Masimo or VSI*] monitoring will alter management of patients in the hospital? How/why not? (CFIR Innovation Recipient/Deliverer Impact)
    *RACE probe: Have you made any clinical decisions based on VSI data trends prior to actual alerts?***
17. **Do you have any additional suggestions or feedback about the [*Masimo or VSI*] monitoring/alerts?**

**Thanks for your time!**
